# Autoactivation of small GTPases by the GEF–effector positive feedback modules

**DOI:** 10.12688/f1000research.20003.1

**Published:** 2019-09-23

**Authors:** Andrew B. Goryachev, Marcin Leda

**Affiliations:** 1 Centre for Synthetic and Systems Biology, Institute for Cell Biology, University of Edinburgh, Edinburgh, EH9 3BF, UK

**Keywords:** small GTPases, Arf GTPases, Rab GTPases, Cdc42, Rho GTPases, cell biology, autoregulation, positive feedback, symmetry breaking

## Abstract

Small GTPases are organizers of a plethora of cellular processes. The time and place of their activation are tightly controlled by the localization and activation of their regulators, guanine-nucleotide exchange factors (GEFs) and GTPase-activating proteins (GAPs). Remarkably, in some systems, the upstream regulators of GTPases are also found downstream of their activity. Resulting feedback loops can generate complex spatiotemporal dynamics of GTPases with important functional consequences. Here we discuss the concept of positive autoregulation of small GTPases by the GEF–effector feedback modules and survey recent developments in this exciting area of cell biology.

Small GTPases, or small GTP-binding proteins, are intimately involved in thousands of vital cellular functions, such as signaling, regulation of cytoskeleton, and membrane trafficking ^1– 4^. The absolute majority of small GTPases undergo nucleotide cycling between active GTP-bound and inactive GDP-bound states. Activity of a GTPase is defined by the ability to interact with its multiple effectors that, upon binding to the GTP-loaded GTPase, frequently undergo activation themselves. Inactive GTPases are found uniformly distributed in the cytoplasm, whereas active GTPases typically reside on the plasma membrane or intracellular membranous compartments. Therefore, activation of a particular GTPase at a specific time and location on a membrane results in both rapid recruitment of its effectors from the cytoplasm and their local activation to enable specific intracellular functions, such as tethering of a secretory vesicle to the plasma membrane or induction of a cellular protrusion. The reverse process of GTPase inactivation causes disassembly of the effector complexes and their recycling back to the cytoplasm. Small GTPases thus play the role of universal licensing factors whose activation enables specific cellular processes. Activity of GTPases is tightly controlled by two opposing families of enzymes—guanine-nucleotide exchange factors (GEFs) and GTPase-activating proteins (GAPs)—that activate and inactivate small GTPases, respectively ^5– 7^. GTPases therefore provide an elegant universal interface between a host of cellular processes that control localization and activity of their GEFs and GAPs and a variety of cellular functions organized by their effectors ^8^. Besides serving as cogs central in the highly interconnected cellular clockwork, small GTPases have a potential for complex emergent behavior. This behavior is possible because GTPases, either via their effectors or directly, can control their own GEFs and GAPs, generating multiple feedback loops ( Figure 1). This feedback enables autonomous dynamics of GTPases, which is no longer dictated solely by the upstream cues. This capability of small GTPases to autoregulate is highly significant as it can potentially explain cell-scale symmetry-breaking events, such as spontaneous cellular polarization ^9^.

**Figure 1.**
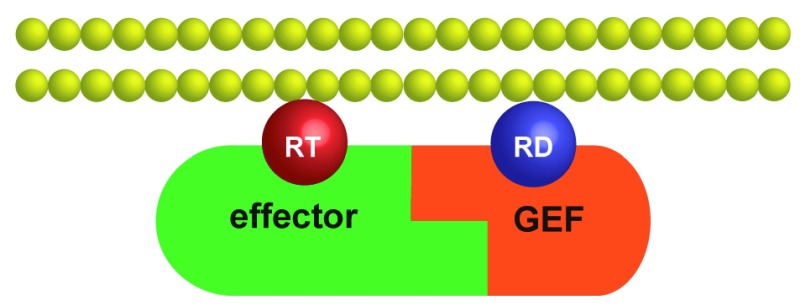
The GEF–effector positive feedback module. The complex between an effector and a GEF is first recruited to the cytosolic face of a membrane by the interaction between the effector and a molecule of activated GTPase, RT. The GEF then can interact with and processively activate inactive GTPase molecules, RD. GEF, guanine-nucleotide exchange factor.

Zerial and colleagues ^10^ provided the first example of a positive feedback loop that controls activity of the Rab family GTPase Rab5 via a complex between its effector Rabaptin-5 and GEF Rabex-5. They also suggested that such a positive feedback loop can form a spatially compact cluster of activated GTPases and thus undermine the spatially uniform state enforced by molecular diffusion. Such an initial symmetry-breaking step provides a prerequisite for cellular morphogenesis. Another positive feedback module based on the Rab family GTPase Sec4 was shown to play an important role in regulating membrane secretion in budding yeasts ^11, 12^. In this module, Sec2, a GEF for Sec4, associates in its phosphorylated form with Sec15, an effector of Sec4 and a subunit of the exocyst complex, which tethers secretory vesicles to the yeast plasma membrane. Similar feedback loops were proposed to activate other Rab and Arf GTPases ^13, 14^. Particularly important for our detailed understanding of this phenomenon has been the paradigm of Ras activation by its GEF SOS (Son of Sevenless) ^15^. Structural analysis determined that SOS can simultaneously bind two molecules of Ras: a GDP-bound Ras via its catalytic domain and active GTP-bound Ras via a nearby allosteric site ^16^ ( Figure 2). Later work demonstrated that binding to active Ras can increase the nucleotide exchange activity of SOS by as much as 500-fold ^17^. While SOS is a complex molecule that interacts also with signaling receptor complexes and membrane lipids, the presence of two independent binding sites, which are specific for the inactive and active GTPases, makes it effectively a fusion between a GEF and an effector. Importantly, the biological significance of Ras activation via the SOS-mediated positive feedback has been experimentally confirmed ^18^. A complementary paradigmatic example has emerged from budding yeast cell biology. Formation of the yeast bud is preceded by the emergence of a plasma membrane localized cluster of activated Rho GTPase Cdc42 ^19^. The molecular underpinnings of this dramatic symmetry-breaking phenomenon have been traced to the formation of a complex between Cdc42 effector Bem1 and GEF Cdc24 ^20, 21^. Theoretical analysis ^22^ demonstrated that Cdc42 polarization belongs to the class of diffusion-driven instabilities of the spatially homogeneous state, which are commonly referred to as the Turing-type pattern-forming instabilities ^23, 24^. Furthermore, modeling ^22^ showed that efficient polarization, associated with the enrichment of Cdc42 in the cluster, requires that, in addition to the Bem1-Cdc24 positive feedback, the membrane-cytoplasmic shuttling of Cdc42 be coupled to its nucleotide cycling. The latter property of Cdc42 is shared by many other Rho and Rab family GTPases whose membrane-cytoplasmic shuttling is mediated by GDP dissociation inhibitor (GDI) proteins that strongly prefer to interact with the inactive GTPases ^26, 27^. Biophysical modeling ^28^ of GTPase activation via the GEF–effector complex found that this mechanism provides both high activity and rapid nucleotide cycling of GTPases, which are observed, for example, in the budding yeast Cdc42 cluster. This study also predicted that positive feedback modules mediated by the GEF–effector complexes should be ubiquitous elements of molecular networks involving small GTPases. However, identification and characterization of the specific molecules involved in these modules turned out to be challenging. Today, more than 20 years after the pioneering discovery of the Rab5–Rabaptin-5–Rabex-5 module, new molecules and interactions continue to be identified whereas functional significance of GTPase autoregulation modules is still far from being understood. Here, we provide an update on recent progress in the quest to understand the behavior and functional significance of these modules.

**Figure 2.**
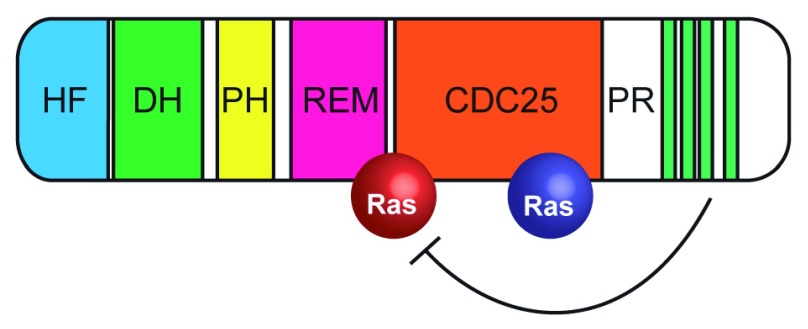
Domain organization and activation of SOS. In the cytoplasm, the proline-rich domain occludes the binding site for Ras-GTP (red). Binding to Grb2 releases this autoinhibition. Domains: CDC25, Ras GEF; DH, Rho GEF; HF, histone fold; PH, pleckstrin homology; PR, proline-rich; REM, Ras exchange. Green stripes indicate PXXP motifs in the PR domain that interact with SH3 domains of Grb2. Inactive Ras molecule bound to CDC25 domain is shown in blue. GEF, guanine-nucleotide exchange factor; SOS, Son of Sevenless. Adapted from 25.

## Autoactivation of Cdc42 in higher eukaryotes: cell polarity revisited

In fungi, Cdc42-GTP forms well-defined polarity clusters that can be readily observed with the use of fluorescent reporters based on the GTPase-binding domains of various Cdc42 effectors. These clusters, found at the actively growing cellular protrusions in yeasts ^29, 30^ and filamentous fungi ^31^, provide a convenient readout that facilitates the analysis of Cdc42 autoactivation dynamics in response to genetic and pharmacological perturbations ^32– 35^. Unfortunately, such conspicuous patterns are not typically seen in cells of higher eukaryotes despite the irrefutable genetic evidence that Cdc42 is crucial for the establishment of cell polarity in all eukaryotes ^36^. Possibly, they will be discovered in the future with the upcoming development of novel reagents and microscopy techniques. Notable exceptions of higher eukaryotic systems that exhibit Cdc42 clusters include early *Caenorhabditis elegans* embryos ^37^ and the enterocyte cell line Ls174-W4 that had been engineered to activate LKB1 kinase in a doxycycline-inducible manner ^38^. The latter system has a striking capacity to develop cell polarity, complete with the brush border, in single cells, rather than in cells immersed in a tissue, as is normally required for cellular polarization in multicellular organisms. The polarized “apical” domain of these cells is enriched in Cdc42. Remarkably, Cdc42 activity was found required for the uniqueness of such a domain ^39^. While owing to the absence of a suitable reporter the activity of Cdc42 could not be visualized directly, a Cdc42 GEF Tuba was implicated in Cdc42 activation ^40^. Earlier, this GEF had been reported to activate Cdc42 at the apical domain of epithelial cells ^41, 42^. Par6 is an effector of Cdc42 that is frequently found in the context of higher eukaryotic cell polarity ^43, 44^. Originally identified in the anterior polarity complex of the *C. elegans* zygote, Par6 was demonstrated to bind activated Cdc42 and thus connect signaling of a small GTPase and the atypical members of the PKC family of kinases, which are recruited by Par6 to the Cdc42-controlled apical domain. Until now, no direct interaction between Par6 and Tuba has been reported. In an exciting development on the theme, Bruurs *et al*. recently reported that Ls174-W4 enterocytes knocked out for Par6 exhibit the same phenotype as the cells depleted of Cdc42 or Tuba ^45^. Indeed, in all these genetic backgrounds, the enterocytes polarize but form multiple disjoint patches of polarity easily detectable by the accumulation of F-actin in the brush border. Importantly, the authors presented evidence that the three proteins—Cdc42, Par6, and Tuba—form a single complex. The abundance of the complex increased with the expression of the constitutively active Cdc42 mutant, confirming that it is the GTP-loaded Cdc42 that is required for the existence of the complex, in complete agreement with the idea of the effector-GEF positive feedback loop based on the interaction between Par6 and Tuba. Remarkably, the data of Bruurs *et al.* suggest that, as in fungi, Cdc42 polarization in cells of higher eukaryotes may also require autoactivation of Cdc42 via a GEF–effector feedback loop.

Another crop of exciting results came recently from a highly complementary study by Elbediwy *et al*. ^46^. These authors started from the interactome of aPKCι, one of the two atypical PKC kinases involved in cellular polarity in the form of the Par6–aPKC complex, which they studied in the human colon cancer cell lines HCT116 and Caco2. They found that aPKCι interacts with and phosphorylates Cdc42 GEFs FARP1 and FARP2. Of these, FARP2, rather than FARP1, appeared to be required for the establishment of polarity in Caco2 cells. As both cell types used in the study were epithelial cells growing in the confluent tissue, activated Cdc42 did not form a polarity cluster but instead appeared at the cell–cell junctions, whose formation and maturation are regulated by Cdc42 ^47^. Interestingly, the authors found that FERM and FERM-adjacent domains of FARPs interacted with RIPR motif of aPKCι. Therefore, the GEF–effector interaction in this system is mediated by aPKCι and thus is indirect. Intriguingly, Bruurs *et al*. ^47^ found that the presence of aPKC within their GEF–effector complex was indeed required for the polarity function, but no specific details of interactions between the molecules were reported. The study by Elbediwy *et al*. ^46^ presents an interesting twist: the phosphorylation of FARPs by aPKCι has a peculiar effect. On the one hand, the authors demonstrated that FARP phosphorylation by aPKCι was required for the junction formation. On the other hand, they found that these phosphorylations disassemble the complex. This puzzling result could be interpreted as an indication that the feedback provided by this complex is mixed and serves to limit excessive activation of Cdc42. Alternatively, if the phosphorylated and presumably activated GEF is “handed over” to other molecule(s) that can retain active FARP at the junctions, this mechanism could promote processivity of FARP activation and thus increase, rather than decrease, the activity of Cdc42 at the junctions. Further work will be necessary to distinguish between these alternatives and clarify the role of aPKCι in the activation of Cdc42.

## SOS: taming autoactivation with autoinhibition

Thanks to the systematic efforts invested over years by the Groves, Kuriyan, Bar-Sagi, and Roose labs, SOS is arguably the most well-studied GEF overall and perhaps also the best understood single-molecule positive feedback module. Yet many questions remained unanswered until very recently. Thus, the true biological function of SOS-mediated Ras autoactivation remained unclear. If SOS is indeed such a potent positive feedback module as the early biochemical analyses implied, why does it not generate micrometer-scale Ras-GTP clusters similar to the clusters of activated Cdc42? Given the potent effect that Ras isoforms exert on cellular proliferation in general and the transforming effect of hyperactivated Ras mutants ^48, 49^, it would be expected that uncontrolled formation of clusters of activated Ras could be very harmful on both cellular and organismal levels. To limit their formation, cells could actively remove such clusters from the plasma membrane where Ras GTPases are typically activated. Indeed, endocytosis appears to be a default cellular response to the formation of signaling clusters at the cell surface. Groves and colleagues recently developed a brilliant *in vitro* reconstitution assay that permits analysis of SOS and Ras activation dynamics with unprecedented precision in time and space ^25^. The assay is performed on a supported lipid membrane microarray ^50^, which is divided into small rectangular cells by a nanofabricated chromium grid. The grid provides a diffusion barrier impenetrable to Ras and other membrane proteins and allows investigators to detect activation of Ras in its individual cells by fluorescence microscopy. The authors found that SOS constructs recruited to the membrane by interaction with lipids and active Ras remained there for many minutes, producing many more active Ras molecules. In order to elucidate how SOS is eventually removed from the membrane, Christensen *et al*. ^25^ turned their attention to the B cell receptor (BCR)-activated cells and found that SOS leaves the membrane in endocytic vesicles together with the activated BCRs. Interestingly, SOS mutants unable to bind activated Ras were removed faster than fully functional SOS.

For a long time, analysis of SOS dynamics *in vitro* was hampered by the challenges with purifying the full-length (FL) protein. As a result, many earlier analyses of SOS activity had been performed with truncated constructs, lacking the N-terminal histone fold (HF) and C-terminal proline-rich (PR) domains, both of which play autoinhibitory roles, independently of each other ( Figure 2). Fortunately, the Groves lab has recently overcome these technical issues ^51, 52^. The study by Lee *et al*. ^52^ with FL SOS extracted from cell lysates demonstrated that the PR domain potently inhibits SOS binding to active Ras on the membrane and thus effectively blocks the engagement of the positive feedback loop. Yet it has long been known that the catalytic activity of SOS not bound to active Ras via its allosteric site is essentially non-existent ^17^. This seeming contradiction is finally resolved by the latest study from the Groves lab who managed a nearly complete *in vitro* reconstitution of Ras activation by SOS, now including phosphorylated LAT and Grb2 adaptor proteins ^51^. This elegant analysis convincingly demonstrated that binding of Grb2 to SOS via the PR domain relieves the inhibitory effect of the latter on the access of Ras to the allosteric site. The authors found a prolonged (50-second) delay between the binding of SOS to the activated receptor complex via Grb2 and noticeable GEF activity of the bound SOS. This suggests that, until one or two Ras molecules are activated by the weak GEF activity of SOS, which is not yet allosterically bound to Ras-GTP, SOS remains in the Grb2-bound but largely inactive state during which it has an opportunity to detach and recycle back to the cytoplasm without generating a potentially unwanted burst of activity. The authors explained this behavior by the principle of kinetic proofreading, which protects the cell from unnecessarily responding to weak sub-threshold signaling stimuli ^53^. Thus, it finally appears that the autoactivation ability of SOS has evolved not to generate spatial clusters, as in the case of Cdc42, but rather to create a built-in safety catch.

## Autoactivation of Arf GTPases

The Arf family of small GTPases ^54, 55^, which are involved primarily with the membrane trafficking between zillions of intracellular compartments, also greatly contributed to the development of the GTPase autoactivation paradigm ^13^. The best understood function of Arf GTPases is the assembly of transport coats, such as COPI and COPII, in which some subunits are Arf effectors ^56^. Jackson and colleagues found that the yeast γ-COP (Sec21) subunit of the COPI coat directly binds Arf1 GEFs Gea1/2 and that their mammalian homolog GBF1 interacts with mammalian γ-COP ^57^. As both complexes were detectable in the absence of Arf1 activity, the authors proposed that these GEF–effector interactions played a role in the recruitment of coat subunits to the membrane rather than in activating Arf1. As other explanations are possible, more work is needed to elucidate the role of these conserved interactions. Apart from these effector-mediated interactions, some Arf GEFs have been shown to directly associate with both active and inactive molecules of the same GTPase. Stalder *et al*. ^58^ reported that the pleckstrin homology (PH) domain of Arf1 GEF Arno (cytohesin-2), a member of the cytohesin family of Arf GEFs, binds not only electronegative lipids but also GTP-loaded Arf6 and Arf1. The latter interaction represents a single-molecule GEF–effector positive feedback module. The authors provided compelling biochemical evidence that positive feedback in this system is highly efficient and likely works via recruitment of Arno to the membrane. Thus, it could result in a spatial clustering of Arf1 and Arno provided that the size of the membrane compartment is sufficient to accommodate this spatially inhomogeneous state. Interestingly, PH domains of several Lbc family Rho GEFs have also been shown to bind active RhoA to generate a positive feedback loop ^59, 60^; however, the functional significance of these interactions remains to be elucidated. Richardson and Fromme identified that yeast Arf1 GEF Sec7 binds to Arf1-GTP via its HDS1 domain ^61^. Like SOS, Sec7, a homologue of mammalian GEFs BIG1 and BIG2, is a complex molecule with multiple molecular interactions and structural domains, some of which inhibit the Sec7 catalytic domain ^62– 64^. Similar to the Arno–Arf1 interaction, binding of the HDS1 domain to Arf1-GTP promotes stable recruitment of Sec7 to the membrane ^61^. Intriguingly, this interaction appears not to be conserved among the higher eukaryotes ^54^. Why it is so remained a long-standing puzzle. Recently, Deretic and colleagues identified an interaction between mammalian GBF1 and Arf4, which is another GTPase activated by this GEF at the Golgi ^65, 66^. As Arf4, which is not present in budding yeast, is partially redundant with Arf1, it is possible that it took over some of the functions of Arf1 that required autoactivation. More work will be needed to better understand the physiological role of Arf GTPase autoactivation in various systems.
